# Modification of an OSCE format to enhance patient continuity in a high-stakes assessment of clinical performance

**DOI:** 10.1186/1472-6920-11-23

**Published:** 2011-05-24

**Authors:** Rose Hatala, Sharon Marr, Cary Cuncic, C Maria Bacchus

**Affiliations:** 1 Department of Medicine, University of British Columbia, Vancouver, BC, Canada; 2 Department of Medicine, McMaster University, Hamilton, ON, Canada; 3 Department of Medicine, University of Calgary, Calgary, AB, Canada

## Abstract

**Background:**

Traditional Objective Structured Clinical Examinations (OSCEs) are psychometrically sound but have the limitation of fragmenting complex clinical cases into brief stations. We describe a pilot study of a modified OSCE that attempts to balance a typical OSCE format with a semblance of a continuous, complex, patient case.

**Methods:**

Two OSCE scenarios were developed. Each scenario involved a single standardized patient and was subdivided into three sequential 10 minute sections that assessed separate content areas and competencies. Twenty Canadian PGY-4 internal medicine trainees were assessed by trained examiner pairs during each OSCE scenario. Paired examiners rated participant performance independent of each other, on each section of each scenario using a validated global rating scale. Inter-rater reliabilities and Pearson correlations between ratings of the 3 sections of each scenario were calculated. A generalizability study was conducted. Participant and examiner satisfaction was surveyed.

**Results:**

There was no main effect of section or scenario. Inter-rater reliability was acceptable. The g-coefficient was 0.68; four scenarios would achieve 0.80. Moderate correlations between sections of a scenario suggest a possible halo effect. The majority of examiners and participants felt that the modified OSCE provided a sense of patient continuity.

**Conclusions:**

The modified OSCE provides another approach to the assessment of clinical performance. It attempts to balance the advantages of a traditional OSCE with a sense of patient continuity.

## Background

High-stakes assessments of clinical performance have significantly improved through the development of tools such as the Objective Structured Clinical Examination (OSCE) [1]. However, the fragmentation of complex clinical cases into brief OSCE stations may result in some loss of validity as the authenticity of comprehensively examining a single patient is lost. Examiners may be constrained from assessing the trainee's understanding of a complex patient [2]. The lack of a full patient assessment may send an inaccurate message to the trainees as to the characteristics of clinical competence valued by the examination board [3].

As an alternative, the traditional long case examination assesses trainees on a whole patient case, using a real patient with problems relevant to clinical practice [4]. However, the measurement properties of a traditional long case examination are poor [2,5].

High-stakes examination boards may struggle when attempting to choose a suitable examination format that meets the psychometric standards of the OSCE and yet includes the patient continuity and steering effect on learners that is present in a long case examination. One potential solution has used work-sampling strategies to assess clinical competence across multiple patients in real clinical practice, as in the mini-clinical evaluation exercise (mini-CEX) [6]. A second solution has been to improve the long case by using direct observation of performance, multiple examiners and standardized rating forms [2,4]. However, for examinations with large numbers of participants, logistical issues may constrain the implementation of these approaches.

The Royal College of Physicians and Surgeons of Canada's (RCPSC) comprehensive examination in internal medicine is a high-stakes examination based on an OSCE format [7]. As members of the examination board, we desired an assessment format that could balance the strengths of an OSCE with a semblance of patient continuity and complexity. We felt that the potentially beneficial steering effect on learners of an examination that emphasized patient continuity and complexity would offset any logistical issues raised by the new format. We describe the development and evaluation of a pilot modified OSCE based on a continuous, complex, patient case.

## Methods

At our national specialty examination, standardized patient (SP) encounters focus on physical examination and communication skills. History-taking skills are not tested in this format. Thus, in our pilot study, history-taking skills were not assessed.

**Figure 1:**
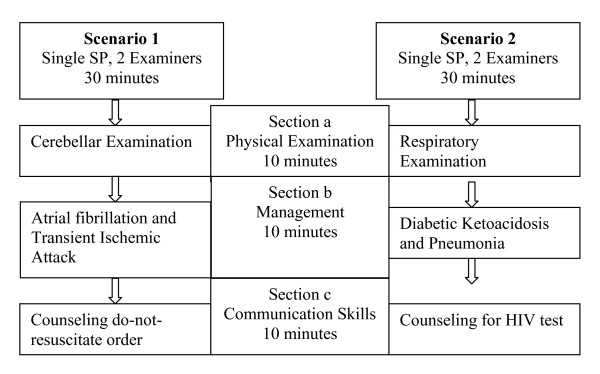
**Format of the modified OSCE**. Each modified OSCE scenario was subdivided into three sequential but independent 10 minute sections. The same SP remained constant throughout a scenario. Participants rotated through both scenarios.

To mimic the sense of continuity present in a typical patient encounter, the modified OSCE consisted of two clinical scenarios, each consisting of a SP and the same two examiners. To maintain content specificity, each scenario was subdivided into three sequential 10 minute sections that assessed separate content areas and competencies (Figure 1). For each scenario, participants began by reading a clinical stem describing the SP's clinical history, performed an observed physical examination, discussed their management strategies with the examiners, and provided observed counseling to the SP. Each section of a scenario sampled a different content area and a separate clinical competency. In order to ensure that participants were not penalized across sections of a scenario, correct answers on a previous section were not required to answer the next section, and each section was scored independent of the previous section. The SPs received three hours of training from one of the study investigators (SM) regarding the clinical details of the scenario.

Participants rotated through both scenarios (Figure 1). For each scenario, participants were rated by two examiners. Following the completion of each 30-minute scenario, participants were provided with verbal feedback. At the conclusion of the modified OSCE, participants and examiners completed satisfaction surveys.

### Study Sites

The modified OSCE was held on two separate dates (5 days apart) at McMaster University and the University of Toronto. Ethics approval was obtained from each of the academic sites.

### Participants

Twenty-four post-graduate year four internal medicine residents were recruited as participants and 20 participated in the study. Participation was voluntary and the participants provided written informed consent. Participants were assigned a unique study number.

### Examiner Training

Fifteen experienced Royal College examiners were recruited nationally (1 examiner participated at both sites). All had experience with the traditional OSCE format. Participants and examiners were unknown to each other and the examiners did not assess the participants at the actual national specialty examination.

Examiners reviewed the OSCE scenarios and participated in a 45 minute training session. During this session, the scenarios and rating scales were reviewed. The importance of scoring each section separately from the previous section and independently from their co-examiner was emphasized. Examples of failing performance as well as passing performance were discussed, although a formal standard setting exercise was not undertaken. The examiners had extensive experience with this rating format as examiner pairs independently rate candidate performance at our national specialty examination.

### Case Development

The two scenarios and their sections were developed by the study investigators and reviewed by members of the RCPSC internal medicine examination board. The clinical scenarios were developed to be sufficiently complex that multiple independent sections could evolve from the initial history.

### Outcome Measures

Participant performance on each section was independently rated using a 7-point global rating scale familiar to the examiners [8]. Ratings of 1-3 constituted failing performance and 4-7 were passing performance.

### Satisfaction Survey

At the conclusion of each OSCE, examiners and participants completed the same satisfaction questionnaire which included 2 five-point rating scales as well as free-response items. The questionnaire asked how well the modified OSCE assessed clinical competence, whether it was more representative of a real patient encounter compared to a traditional OSCE format, and which OSCE format best assessed clinical competence.

### Statistical Analysis

Descriptive statistics were used for group performance data and the satisfaction questionnaire ratings. Inter-rater reliabilities for each of the ratings were calculated using an intra-class coefficient. Average ratings across examiner pairs were used for subsequent analyses and a mean cut-point score of 3 was used to classify performance as pass or fail. A 2X3 repeated measures analysis of variance was conducted to examine the effect of scenario and section on participant's performance ratings, with scenario (scenario 1, scenario 2) and section (section a, section b, section c) treated as within-subject factors. The study was underpowered to undertake an analysis by study site. A generalizability study to determine the reliability of the examination was conducted [9]. Pearson correlations between sections (both within and across scenarios) were calculated.

Descriptive statistics and correlations were performed using SPSS 15.0. G-string [10] and UrGenova [11] were used to conduct a generalizability analysis (G-study) and a decision study.

Two of the authors (RH and CC) independently classified the free-response comments from the questionnaire into themes. Common themes are presented and representative participant statements are included in italics.

## Results

One participant was missing ratings from both raters on a section and therefore the results for this participant were removed from the analysis.

### Descriptive Statistics

There was no main effect of section (F(2,36) = 0.93, p = .41) or scenario (F(1,18) = 1.26, p = .28), indicating that a participant's performance did not vary significantly by section or scenario. Participants' mean ratings per section are shown in Table 1. Across the six sections, 5 participants passed all sections, 6 participants failed one section, 3 participants failed 2 sections, 2 participants failed 3 sections, 1 participant failed 4 sections and 2 participants failed 5 sections.

**Table 1:** Participants' mean ratings per section

	Mean Rating	Standard Deviation	# Participants passing the section
Scenario 1, Section a	4.11	1.23	13/19

Scenario 1, Section b	4.46	1.05	18/19

Scenario 1, Section c	4.67	0.81	18/19

Scenario 2, Section a	4.32	0.81	16/19

Scenario 2, Section b	4.21	0.79	17/19

Scenario 2, Section c	4.17	0.65	18/19

Performance on each section was independently rated by 2 trained examiners using a 7-point global rating scale [8]. Ratings of 1-3 constituted failing performance and 4-7 were passing performance.

### Variance components analysis (G-study)

Although rater was originally a facet of this analysis, on occasion raters did not assign a rating on particular sections. To keep a balanced design, it would have been necessary to remove data for these participants but this would have reduced the number of participants in the model. We decided to average the ratings for each pair of raters to produce section scores and thereby allowing us to use the data from all the participants. The results of the generalizability study are shown in Table 2. The component that accounted for most of the variability in scores was the residual error term. This term represents both the variability in the ratings due to the interaction of participants, scenarios and sections and any variability that was unaccounted for in the design. The next highest component was that associated with participants, indicating that there were differences between the scores assigned to the participants. This confirms the intent of the assessment, which was to discriminate between participants' performances. The interaction between participants and scenarios suggests that it would take several scenarios to produce a reliable score. The remaining components did not contribute much toward the variability on the ratings.

**Table 2:** Variance components of the participants' ratings

Sources of variance	Variance component	% variance
Participant	0.298	34

Scenario **	0	0

Section **	0	0

Participant X Scenario	0.138	16

Participant X section	0.036	4

Scenario X section	0.044	5

Participant X Scenario X section*	0.357	41

* residual error term.

** These components were negative and reset to zero for reporting purposes and for generating the g-coefficient.

To determine the reliability of the scores on the examination, the variance components were used to calculate a g-coefficient for the examination using the following formula:(1)

in which n_s _= the number of scenarios (2), n_i _= the number of sections (3), and n_is _= the number of scenarios × sections (6). The g-coefficient for this model is 0.68. Based upon this model, it would require four scenarios to achieve a g-coefficient of 0.80. Increasing the number of sections per scenario would have little effect.

### Pearson correlation coefficients

There was moderately high correlation between performance within sections of a single scenario with correlations ranging from 0.55 to 0.69 (all p-values < 0.05), aside from one correlation of 0.20 between the management and patient counseling sections of Scenario 1 (p = 0.36). Correlations between sections across the two scenarios ranged from 0.15 to 0.65.

### Inter-rater reliability

Although there were data missing from the sections for several participants that prevented rater from being included in the generalizability analysis, we were interested in determining the inter-rater reliability of the sections. An intra-class correlation was determined for each section within each scenario. The inter-rater reliabilities ranged from 0.62 to 0.91 on scenario 1 and 0.73 to 0.88 on scenario 2 indicating a relatively high level of agreement between the rater pairs.

### Perceptions of the OSCE

Eighty percent of examiners, and 85% of participants agreed or strongly agreed (on a 5 point rating scale) that the modified OSCE allowed the participants to demonstrate their clinical competence. Compared to a traditional OSCE, 60% of examiners and 65% of participants agreed that the scenarios were more representative of a patient encounter. Among examiners, 53% felt the modified OSCE was a better assessment of clinical competence, 7% felt traditional OSCE stations were better, and 40% of examiners were unsure. Fifty-five percent of participants thought the modified OSCE scenarios were a better assessment of their clinical competence, 30% preferred a traditional OSCE and 15% participants were neutral or unsure.

Two main themes emerged from the written comments: realism and the assessment format. Compared to a traditional OSCE, both examiners and participants thought that the modified OSCE was more realistic of a true clinical situation, *"It felt more real"*, and that the sections flowed more naturally. The examiners' comments indicated that they were equivocal as to what the modified format added to a traditional OSCE: *"... unsure whether these are really 'long' cases" *whereas the candidates seemed to feel it was a *"better assessment of overall knowledge"*.

## Discussion

The intent of this pilot study was to examine the modified OSCE, using generalizability theory to inform an understanding of this approach in a high-stakes assessment of clinical performance. The concept of linked OSCE stations is not new, however we are unaware of research that has formally assessed the psychometrics of such an approach. As outlined in a recent review article, the disadvantages of the long case format limit its utility in high-stakes assessment [12]. Our modified OSCE addressed three limitations commonly found in long case assessments: a) sampling of multiple content areas; b) assessments by multiple examiners; c) sampling of multiple competencies [2]. Our modified OSCE format attempted to balance content specificity with a sense of patient continuity.

Examining the sources of validity evidence [13], the modified OSCE sampled across multiple content areas relevant to internal medicine. The response process was based on a previously validated rating scale [8]. The psychometric data demonstrated that the trained examiners had acceptable inter-rater reliability and that 4 scenarios would meet the standard of a g-coefficient of 0.80 for a high-stakes examination purpose [14]. The consequences of the assessment tool were examined qualitatively, with the majority of examiners and participants supporting the modified OSCE as a reasonable clinical performance assessment tool.

There are limitations to the current study. We are unable to comment on the representativeness of our participant sample, as we did not have access to other measures of their clinical performance aside from their study data. As this was a pilot study, the sample size was small and additional larger studies of this format will be necessary. The small sample size and the use of only two scenarios results in variance components that would have wide confidence intervals. However, the advantage of an approach based on generalizability theory is that it permits examination of the implications of increasing or decreasing the sources of the variance. Based on the pilot data, increasing the number of scenarios to 4 could yield a high-reliability assessment.

As the stimulus for the pilot study came from our national specialty examination, we mimicked the format of that examination and did not include an assessment of history-taking skills. The authenticity of the SP encounter would benefit from including history-taking.

With the modified format, we attempted to strike a balance between content specificity and a sense of patient continuity. We observed higher correlations among sections within a scenario compared to sections across the two scenarios. Despite having carefully constructed the scenarios so that correct answers on each section did not influence performance on the subsequent sections, the higher within-scenario correlations suggest that there was a loss of content specificity. Alternately, this observation could be due to a halo effect within each scenario, as each examiner pair rated a participant's performance across all 3 sections of a scenario[15]. Future studies should use separate examiners for each section to eliminate any halo effect. Even if a halo effect is operative, a modified OSCE containing 4 scenarios may achieve acceptable reliability.

The examiners' comments pertaining to the realism and assessment format of this modified OSCE raise some interesting possibilities for future research. It is perhaps a difficult goal to achieve both patient continuity and rigorous psychometrics using only one examination format. A combined examination that included both modified and typical OSCE stations would be one potential approach that capitalizes on the advantages of each format.

## Conclusions

The pilot data suggest that the modified OSCE may provide another approach to the assessment of clinical performance. This format attempts to balance the content specificity advantages of a typical OSCE with a sense of continuous, complex, patient. The higher correlations among sections within scenarios in this preliminary study indicates that this balance may have swung too far in favour of patient continuity. Nevertheless, the positive response expressed by both examiners and participants suggests that negotiating this balance between content specificity and patient continuity in OSCE formats deserves further exploration.

## Competing interests

The authors declare that they have no competing interests.

## Authors' contributions

RH designed the study, participated in data analysis and interpretation, and drafted the manuscript. SM designed and co-ordinated the study, participated in data analysis and interpretation, and reviewed the manuscript. CC participated in designing the study, assisted with study co-ordination, participated in data analysis and interpretation and reviewed the manuscript. CMB participated in designing the study, assisted with study co-ordination, participated in data analysis and interpretation and reviewed the manuscript. All authors read and approved the final manuscript.

## Pre-publication history

The pre-publication history for this paper can be accessed here:

http://www.biomedcentral.com/1472-6920/11/23/prepub
